# Case Report: Double-Decker Repair of Partial Pulmonary Venous Return Into the Coronary Sinus

**DOI:** 10.3389/fcvm.2022.853005

**Published:** 2022-04-05

**Authors:** Xuan Jiang, Jinduo Liu, Yueheng Liu, Tianxiang Gu

**Affiliations:** Department of Cardiac Surgery, First Affiliated Hospital, China Medical University, Shenyang, China

**Keywords:** partial anomalous pulmonary venous return, persistent left superior vena cava, congenital heart disease, cardiac surgery, pulmonary vein

## Abstract

We present a case of persistent left superior vena cava (LSVC) draining into the right atrium (RA) *via* the coronary sinus (CS), while the left superior pulmonary vein returns abnormally to the CS. The LSVC may have few clinical consequences but complicates surgical repair of partial anomalous pulmonary venous return (PAPVR). Transthoracic echocardiography and computed tomographic angiography (CTA) showed that a persistent LSVC and PAPVR converged behind the left atrium. During the operation, the left atrium was adjacent to the confluence part. We resected a portion of the adjacent left atrium to create an inlet of the pulmonary veins and used two autologous pericardial patches to reconstruct a tunnel directing flow from the left pulmonary veins to the surgically created inlet in the adjacent left atrium, and another upper tunnel directing flow from the LSVC to the dilated CS. Pulmonary CTA confirmed that both PAPVR flow to LA and LSVC flow to RA were unobstructed. At a 12-month follow-up, the patient was asymptomatic. No supraventricular arrhythmia was detected. We would like to present this additional technique to our armamentarium to treat PAPVR in combination with LSVC.

## Introduction

Partial anomalous pulmonary venous return (PAPVR) is the most common type of pulmonary venous return anomaly (1). The surgical procedure for PAPVR drainage into the coronary sinus (CS) is controversial, especially in conjunction with a left superior vena cava (LSVC) draining to an intact CS. The combination of these two congenital defects was managed by using two patches to restore the LSVC to the CS in combination with the restoration of normal connection of pulmonary venous drainage to the left atrium in this case report.

## Case Description

A 27-year-old female patient presented to our institution due to palpitations for 2 months. The patient has no history of shortness of breath or fatigue. She gave birth to a child 5 months ago. During pregnancy, her heart palpitations were very severe, and diuretics could partially relieve her. At the initial visit to cardiac surgery, her height was 164 cm, and weight was 60 kg. Her blood pressure, heart rate, and respiratory were normal. This patient has no murmur.

**Figure 1:**
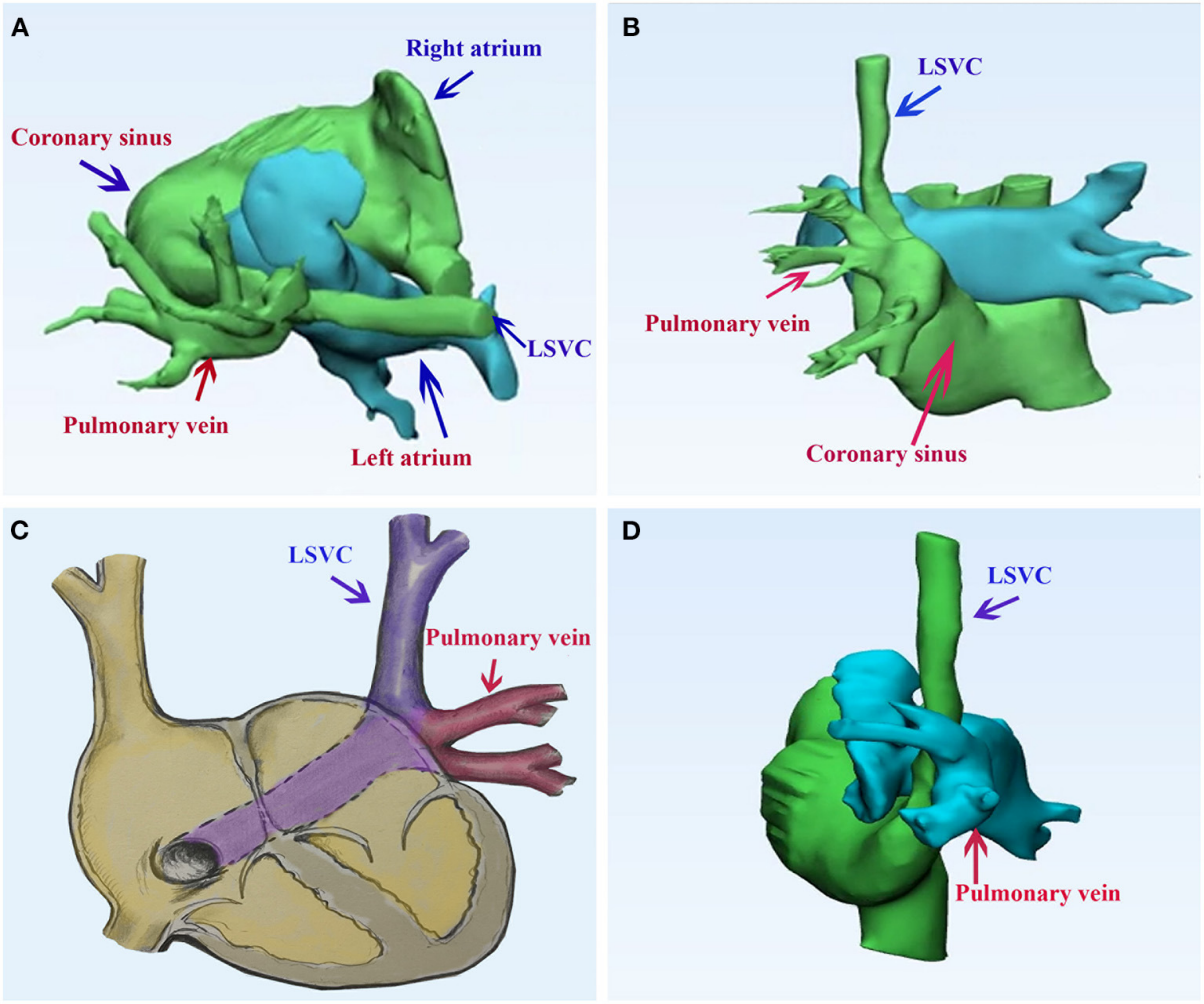
Pulmonary computed tomography of the case of persistent left superior vena cava draining into the right atrium *via* the coronary sinus, while the left superior pulmonary vein returns abnormally to the coronary sinus. **(A,B)** The 3-dimension model of the congenital heart disease; **(C)** a diagram of the case; **(D)** the postoperative 3-dimension model of the double-decker technique. LSVC, left superior vena cava.

Transthoracic echocardiography showed a persistent LSVC draining into the CS and anomalous left pulmonary veins draining into the intact and dilated CS. LSVC and PAPVR converged behind the left atrium. Bilateral superior vena cava of comparable size can be seen. We could not detect the innominate vein. The right heart structure was dilated. Mimics software (20.0, Materialise, Belgium) was used to reconstruct the 3-dimension of congenital heart disease (Figure 1). According to the 3-D model, the left atrium is adjacent to the confluence of LSVC and PAPVR.

**Figure 2:**
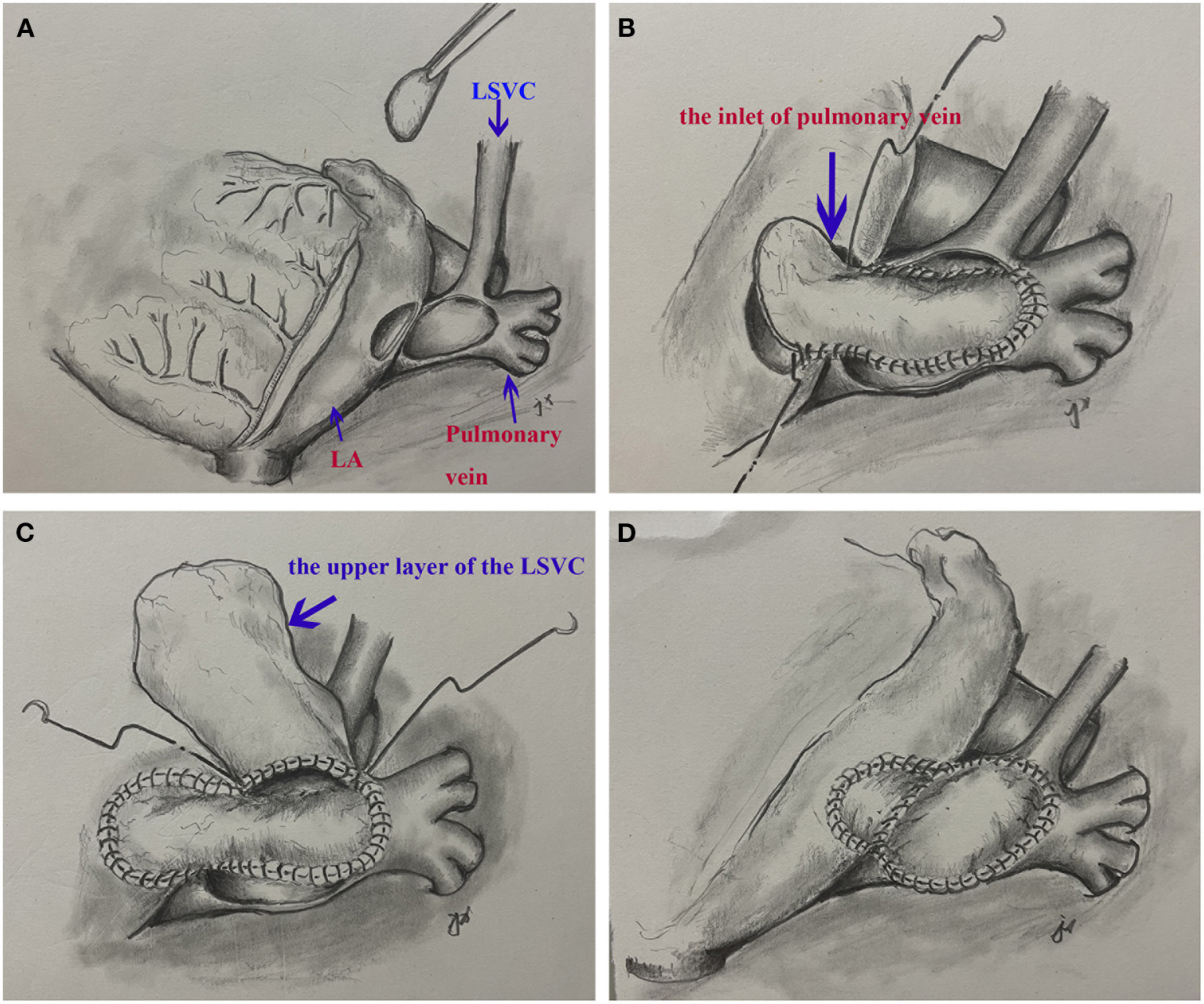
A surgical view of the double-decker technique. **(A)** Resection of a portion of left atrium and cut the confluence of the left superior vena cava and pulmonary veins. **(B)** The autologous pericardial baffle is sutured by guiding the blood flow of the left pulmonary vein to the surgical entrance of the adjacent left atrium. **(C)** The other pericardium creates another upper tunnel, directing blood flow from the left superior vena cava to the dilated coronary sinus. **(D)** The completed double-decker technique.

The patient underwent surgical repair. Standard median sternotomy was performed. Cardiopulmonary bypass was established using standard ascending aortic and bicaval cannulation. After the heart was arrested, a persistent LSVC cannulation was inserted through the enlarged CS. The confluence of LSVC and PAPVR was explored and detected. The left atrium was adjacent to the confluence part. We resected the partial adjacent left atrium (about 2^*^3 cm) to create an inlet of pulmonary veins. A surgically created tunnel was formed from a baffle of the autologous pericardium that is sewn inside the left pulmonary vein in such a way as to direct flow from the left pulmonary veins to the surgically created inlet in the adjacent left atrium. The LSVC was left there. Then, we used another pericardium to create another upper tunnel to direct flow from the LSVC to the dilated CS (see Supplemental Material and Figure 2), which overrode the reconstructed pulmonary vein. The reconstruction tunnel that leads the left pulmonary veins to the left atrium is located below the new tunnel that leads the LSVC to the CS. The inferior pericardium was shared as part of the ventral wall of the LSVC channel and the dorsal wall of the pulmonary venous channel. We used 6/0 Prolene on the 8-mm needle to suture the natural pericardial patch and took care to avoid the narrowing of the two tunnels during the operation. Pulmonary CTA confirmed that both PAPVR flow to LA and LSVC flow to RA were unobstructed (see Figure 1). The pulmonary venous drainage was widely patent. At a 12-month follow-up, the patient was asymptomatic. No supraventricular arrhythmia was detected.

## Discussion

PAPVR represents a physiological left-to-right shunt, which may increase the subsequent risk for pulmonary vascular disease, biventricular failure, and Eisenmenger syndrome (2). Surgical correction of PAPVR is commonly carried out using internal patch technology or its modification and the Warden procedure (4). By baffling these structures with pericardium or a bovine patch, performing or without patch cavity angioplasty as needed, thereby redirecting abnormal pulmonary venous return through the intra-atrial defect. However, when there is no atrial defect and LSVC is present, the best surgery for a complete repair cannot be determined.

The incidence of persistent LSVC is ~0.3%, and the incidence of PAPVR is 0.6–0.7% (3). We can only identify one report on this rare combination of systemic and pulmonary venous anomalies, which together drainage into the CS (3). This combination complicates the repair of PAPVR. Ligating the LSVC may be one choice if tolerated; we had carefully measured the diameter of the LSVC and examined the CTA before the procedure. We found the LSVC was similar in size to the right superior vena cava, and there was no decompressing bridging vein. Ligation of the LSVC may lead to venous congestion and possible intracerebral hemorrhage if there is no bridging vein or the vein is of insufficient size. Extracardiac techniques involving transposition of the LSVC to the right atrial appendage and unroofing of the CS to the LA are an alternative method, just like the modified Warden procedure on the right side. The Warden procedure was used to deal with multiple-level entry pulmonary veins, without transecting or manipulating the cavoatrial junction. For right-sided PAPVR, this approach reduces the incidence of sinus rhythm loss but carries the risk of SVC obstruction. Insufficient length of LSVC is a critical limitation of adult extracardiac technology. We measured the length of the LSVC and found that the LSVC was not long enough to connect directly to the right atrial appendage. Furthermore, we have to perform an overall dissection of LSVC, which is somewhat complex and time-consuming compared to our technique. Also, the deoxygenated coronary venous return to the LA was inevitable *via* intra-atrial baffling. Another alternative method is to tunnel the LSVC to the right on the roof of the atrium. It can also achieve the surgical treatment aim of PAPVC draining into the right atrium: complete correction by rerouting the abnormal pulmonary veins to the left atrium and redirecting the LSVC to the right atrium. Due to the relatively large size of LSVC, the tunnel should be created carefully without obstruction. By using our method, there is no residual abnormal systemic drainage, and no intra-atrial manipulation is required. An alternative approach is to isolate the left pulmonary vein (with the resulting defect patch) followed by anastomosis with the left atrium/left atrial appendage, which seems simpler. However, we found insufficient tissue for direct anastomosis. Direct anastomosis may also lead to subsequent pulmonary vein stenosis and LSVC compression. We also avoid the end-to-end anastomosis between the LSVC and the RAA and decrease the risk of late stenosis of the systemic venous chamber.

To facilitate unobstructed LSVC drainage and right pulmonary vein drainage is critical. For drainage of the right pulmonary vein, we preserved the natural venous tissue underneath to preserve the growth potential and prepared the appropriate pericardial patch to create a tension-free tunnel and prevent late pulmonary vein stenosis. Nevertheless, pulmonary venous stenosis may develop due to the shrinkage of an untreated autologous pericardial patch used to baffle the venous drainage (4). It still needs further exploration.

Double-decker repair is introduced by Hongu (5). The author developed a new surgical technique with minimum right atriotomy and double-barreled arrangement of systemic and pulmonary venous channels. In his report, pulmonary venous blood flows through the proximal SVC, intra-atrial tunnel, and venous sinus defect into the left atrium. Systemic venous blood flows through the ventral opening of the SVC and the RAA chicane that runs across the proximal SVC into the RA. He also created a surgical ASD in patients with an intact atrial septum. Our method is similar to his previous report. But due to the different anatomical entities, our method is easier to understand. The direction of the reconstructed tunnels is parallel to the original vessels. We speculate that our technique would reduce turbulence and achieve good long-term results.

Supraventricular arrhythmia following surgical repair is an important and distressing feature (4). But most of it occurs in patients who have incisions or suturing through the anterior right atrial-superior vena cava junction, which can lead to the risk of sinus node dysfunction. For our technology, we speculate that any type of arrhythmia is less likely to occur. In addition, postoperative anticoagulation is not required.

After all, the advantages of our technique are numerous: (1) the integrity of the left atrium, right atrium, and atrial septum was preserved; (2) both tunnels retained growth potential; (3) anastomosis and manipulation of the entire circumference of the LSVC and PAPVR are avoided; (4) there is no residual anomalous pulmonary or systemic drainage. The Warden, single-patch, double-patch, and double-decker techniques (5) are complementary options for the treatment of PAPVR, and the choice depends on the location of the sinus node, the extent of the right atrium, the location of the pulmonary vein, the size of SVC, and so on.

## Data Availability Statement

The original contributions presented in the study are included in the article/Supplementary Material, further inquiries can be directed to the corresponding author/s.

## Ethics Statement

Written informed consent was obtained from the individual(s) for the publication of any potentially identifiable images or data included in this article.

## Author Contributions

All authors listed have made a substantial, direct, and intellectual contribution to the work and approved it for publication.

## Conflict of Interest

The authors declare that the research was conducted in the absence of any commercial or financial relationships that could be construed as a potential conflict of interest.

## Publisher's Note

All claims expressed in this article are solely those of the authors and do not necessarily represent those of their affiliated organizations, or those of the publisher, the editors and the reviewers. Any product that may be evaluated in this article, or claim that may be made by its manufacturer, is not guaranteed or endorsed by the publisher.

## Supplementary Material

Supplementary Video 1 The procedure of the double-decker technique.
